# Supplementary material for: Promotion of Plant Growth in Arid Zones by Selected Trichoderma spp. Strains with Adaptation Plasticity to Alkaline pH
Source: Biology (Basel). 2022 Aug 12;11(8):1206. doi: 10.3390/biology11081206 (PMC9405189; doi:10.3390/biology11081206)
Supplement: Supplementary file 1 [file biology-11-01206-s001.zip › biology-1697381-supplementary.pdf]

Table S1: Primers used as molecular markers for fungi

Code	Sequence 5´ - 3´	Reference
EF1 728f	CATCGAGAAGTTCGAGAAGG	[29]
EF1 1281r	AACTTGCAGGCAATGTGG	[29]
ITS 1	TCCGTAGGTGAACCTGCGG	[47]
ITS 4	TCCTCCGCTTATTGATATGC	[47]
LROR	ACCCGCTGAACTTAAGC	[48]
LR5	TCCTGAGGGAAACTTCG	[48]
Bsens	ATCACWCACTCICTIGGTGGTGG	[48]
Brev	CATGAAGAARTGIAGACGIGGG	[48]
ACT 512f	ATGTGCAAGGCCGGTTTCG	[49]
ACT 783r	TACGAGTCCTTCTGGCCCAT	[49]
